# Navigating qualitative methods choices in dissemination and implementation research

**DOI:** 10.1186/s13012-025-01476-x

**Published:** 2025-12-17

**Authors:** Jodi Summers Holtrop, Brooke Dorsey-Holliman, Alison B. Hamilton

**Affiliations:** 1 Department of Family Medicine, School of Medicine, University of Colorado, Anschutz Medical Campus, Aurora, CO 80045 USA; 2 Adult and Child Center for Outcomes Research and Delivery Science, School of Medicine, University of Colorado, Anschutz Medical Campus, Aurora, CO 80045 USA; 3 VA Center for the Study of Healthcare Innovation, Implementation, & Policy, VA Greater Los Angeles Healthcare System, Los Angeles, CA 90073 USA; 4 Department of Psychiatry and Biobehavioral Sciences, David Geffen School of Medicine, University of California Los Angeles, Los Angeles, CA 90025 USA

**Keywords:** Qualitative methods, Dissemination and implementation

## Abstract

Qualitative methods are critical to the conduct of Dissemination and Implementation (D&I) research because they illuminate processes, relationships, contexts, and other phenomena known to influence implementation and dissemination. Given the multitude of methods available, choosing appropriate and feasible methods can be challenging, leading many to rely on a limited set of methods. Navigational assistance with methods decision-making, including choosing to use less common methods, is lacking. This paper outlines how to select study methods, beginning with the research goal and the type of research question(s), and presents methods options based on key characteristics of the research. Decision pathways and considerations important to decision-making are featured as well as brief descriptions of the main methods available. Examples are also presented for instructional purposes. This paper supports the field of D&I by addressing a gap in the existing literature about how to conduct qualitative methods D&I research from a methodological perspective.

Contributions to the literature

- There is a need for guidance about how to select and apply appropriate qualitative methods in Dissemination and Implementation (D&I) research. This paper addresses that gap and describes a broader repertoire of methods choices than researchers may have previously considered.
- This paper describes decision pathways with examples corresponding to key issues in D&I research.

## Introduction

Dissemination and implementation (D&I) research is characterized by its focus on supporting the translation of evidence-based interventions into practice. Studying this translation requires knowledge and use of many different research methods, including qualitative, quantitative, and mixed [1]. Methods, informed by theories, models and frameworks (TMFs), support exploration into real-world problems and advance understanding of translational research. Study design and methods innovations have flourished in D&I research given the need to “speed up” the trajectory from basic science to implementation and dissemination [2–6].

As D&I methodologists who teach and provide consultation services to people seeking assistance in conducting D&I studies, we often receive inquiries about choices regarding study design and methods, particularly qualitative and mixed methods. We have observed three key challenges amidst these inquiries. First, many researchers, especially those new to research and/or to D&I, rely on commonly used qualitative methods (e.g., focus groups) and do not tend to use a broad range of methods [7]. Thus, an overview of a broader range of methods, and combinations of methods, is warranted. Second, while many sources of information are available about qualitative methods (e.g., individual interviews, focus groups), these resources often explain each individual method, but do not necessarily support decision-making related to which methods to use and how to combine them, i.e., they typically do not spell out how to start with a research question and then take the next decisional steps toward putting the research plan together. Third, many core methods texts lack specificity for D&I science; adapting methods to this field is an important differentiating factor in choice of qualitative methods and how they may be used [5, 8]. Further, our experiences with teaching research methods to D&I students revealed this dilemma of how to apply methods in the context of D&I studies, which are often complex and fast-paced, and involve multiple disciplines.

Without intentional methods decision-making, D&I studies may not fully capture the complexity of the phenomena under study, which may blunt potential learning through D&I research. Therefore, this paper provides guidance regarding methods appropriate to D&I, when different methods may be appropriate for consideration, and how methods may be combined. We will provide examples and possible navigational paths for different types of questions in D&I research. In this paper we extend the knowledge and use of common D&I qualitative methods outlined in Hamilton and Finley [5].

## Background

### Qualitative and mixed methods

Qualitative research is within a domain of research that produces findings without reliance on quantitative factors or calculations, or most often thought of as words, not numbers [9]. Qualitative methods are critically important to the study of both dissemination and implementation—including but not limited to the development or adaptation of interventions and/or the selection and use of implementation strategies [10]—because they illuminate the who, how, and why in a way that is often missing in quantitative research [1, 5]. D&I research examines not only clinical or health outcomes but also implementation outcomes such as feasibility, acceptability, adoption, penetration, and sustainability [11, 12]. Additionally, as context is a key component of D&I research, qualitative research is especially adept at capturing nuances about the setting and circumstances in which important features about implementation are revealed [5, 13–15].

Mixed methods are also critically important to D&I science, involving integration of the two types of methods in ways that may be sequential (i.e., quantitative to qualitative or qualitative to quantitative) or parallel (i.e., quantitative and qualitative at the same time) or combined in other, more complex designs [16]. Integrated mixed methods can involve data transformation from one form to another (qualitative to quantitative or the reverse) [17]. Mixed methods have the potential to create even more knowledge from existing data sources by the synergy of the integration process. Like qualitative methods alone, mixed methods help researchers to expose and understand the processes and outcomes of an intervention or implementation or dissemination strategy. While an extensive discussion of mixed methods is beyond the scope of this paper, many excellent references exist [16, 18–21].

### D&I theories, models and frameworks

An important starting point in selecting methods is to identify first a theory, model, and/or framework (TMF) that best supports the research question. D&I science is characterized by many TMFs so that research can be organized in a way to clarify the processes, determinants, and effects of different aspects of an intervention. For example, the RE-AIM model seeks to separate out the reach and effectiveness (R and E) of an intervention at the patient- or participant-level outcomes; whereas, the AIM parts—adoption, implementation and maintenance—focus on the setting or intervention agent outcomes [22, 23]. Some TMFs focus on outcomes such as RE-AIM, while others focus on process such as the Exploration, Preparation, Implementation, Sustainment (EPIS) Framework, [24] and still others focus on determinants influencing outcomes such as the Practical Robust Implementation and Sustainability Model (PRISM) [13, 25] or Consolidated Framework for Implementation Research (CFIR) [26, 27]. The purpose of this paper is not to provide an in-depth explanation of any one TMF, but rather to consider what the selected TMF is intended to accomplish within methods decision-making. For example, RE-AIM domains could be considered quantitatively, qualitatively, or through mixed methods [28]. We encourage researchers to visit dissemination-implementation.org for further information and guidance for selecting TMFs for D&I research [29–31].

## Research questions and study design

**Fig. 1:**
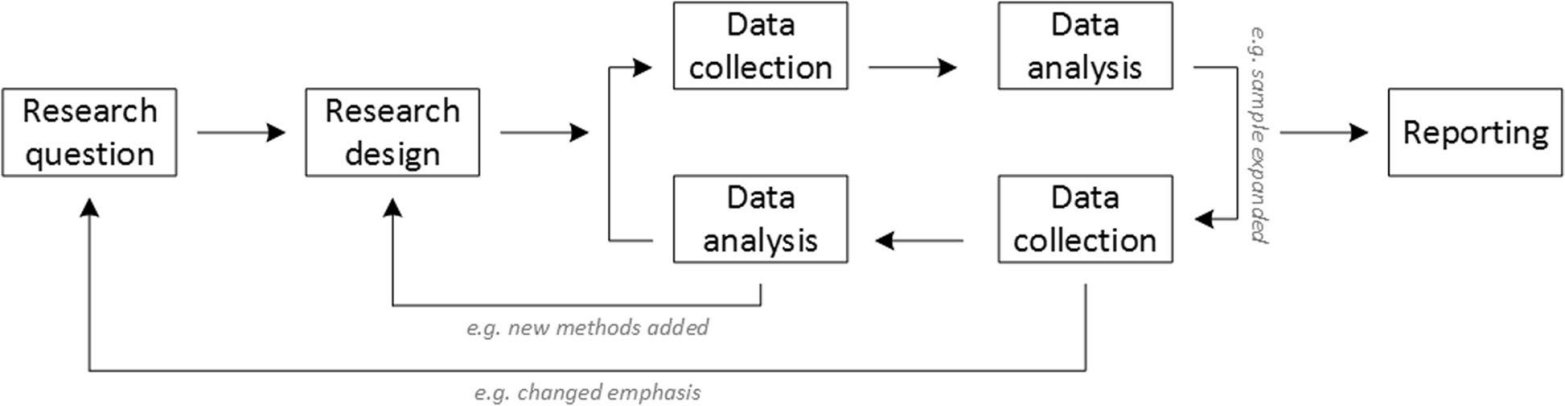
Research Process. Reprinted from Busetto L, Wick W, Gumbinger C. How to use and assess qualitative research. methods. Neurol Res Pract. 2020;2:14. doi:10.1186/s42466-020-00059-z. https://neurolrespract.biomedcentral.com/articles/10.1186/s42466-020-00059-z. Under Creative Commons CC BY 4.0

Although the focus of this paper is not on how to design effective research questions nor on all possible study designs, consideration of methods is intermingled with these topics. Figure 1 outlines an approach to consideration of methods, in the pathway of the research question(s) and the designs chosen [32].

A key starting point is the research question. Good research questions have complex answers but need to be focused, specific, and original, or FINER (feasible, interesting, novel, ethical, and relevant) [33]. They should be answerable within the specified timeframe and with the relevant, appropriate resources. Good research questions that guide design and methods decisions usually start with “how” or “what.” In formulating a research question, it can be helpful to consider the conclusion that you hope to support or the goal you hope to achieve, and then work back to the steps—and question(s)—that will lead there [34]. Often we start with questions that are too broad. “Pruning,” or cutting away anything that isn’t necessary, can be accomplished by considering what knowledge gap you want to fill, what needs to change, and what results would potentially look like [34]. A rubric such as PICOT—Population, Intervention, Comparator, Outcome, Timeframe—is useful for pragmatic designs that are popular in D&I science [35].

Per Fig. 1, the research question drives the selection of the research design. The research design addresses and operationalizes the research question and shapes methods decision-making. D&I research designs are outlined in these resources [3, 36, 37] but commonly include experimental designs involving randomization as well as quasi-experimental and observational designs and even newer adaptive designs or approaches such as SMART [38] or MOST [39]. Qualitative and mixed methods can be useful across these array of designs in the form of implementation evaluations, from exploring the area of inquiry such as with an experimental design to answer such questions as “why did we get the results we did?” or an observational design that may explore “what is important context to this problem?” or “what might facilitate or impede dissemination of efforts to address this problem?” These methods can also be useful for understanding specific interventions, implementation or dissemination strategies, or both with questions such as “how did the participants react to the intervention?” or “how did the implementation strategy work in practice?” Mixed methods can be particularly useful in untangling threads of findings across different data sources to expose the realities of implementation or dissemination. Common mixed methods designs include convergent parallel, explanatory sequential, exploratory sequential, embedded, and transformational [40]. These designs allow variations in which comes first (qualitative or quantitative) or at the same time, or if there are phases to the mixed methods integration.

### Qualitative interpretive frameworks

Interpretive frameworks—not to be confused with TMFs—are essential tools in qualitative research, providing a lens through which researchers can collect, analyze, and understand their data. These frameworks guide the interpretation of findings and analytic objectives, ranging from descriptive to deeply interpretive goals [41]. Some commonly used qualitative interpretive frameworks, sometimes referred to as epistemologies or paradigms, include: phenomenology, grounded theory, ethnography, case study, narrative analysis, and discourse analysis [42]. Qualitative descriptive approaches have been encouraged in health services research [43] and are emerging in implementation science, [44] but historically have been undervalued and critiqued for not being sufficiently interpretive [45].

Interpretive frameworks are a new topic within D&I research and are often not utilized, at least not in the ways we traditionally use them within qualitative research. Qualitative D&I-related analysis often calls upon some form of thematic or constant comparative analysis, and increasingly rapid analysis, [46] but other options are available, such as pragmatic approaches to combining techniques, [47] and may provide opportunities for alternate interpretations [48]. Interpretive frameworks such as those mentioned above (e.g., phenomenology) may add more insight given their focus on other aspects of the work. However, we posit that perhaps the strong emphasis on D&I-specific TMFs (as discussed above) essentially serves some of this purpose by doing what many interpretive frameworks hope to accomplish, i.e., ensuring that the analysis is systematic, rigorous, and meaningful, allowing researchers to draw well-founded conclusions and contribute to the broader field of study within an appropriate contextual domain.

As expected, the choice of interpretive framework alone or in combination with a D&I TMF depends on the research questions, objectives, and the nature of the data. An interpretive framework could be combined with other TMFs, such as utilizing a focused ethnographic approach while drawing on a range of theories as “sensitizing concepts,” [49] or it could be used on its own, such as to understand mental models, [50] for example of clinical practice guidelines and implementation strategies using grounded theory techniques [51]. Desveauz and colleagues used Normalization Process Theory to guide data collection and reflexive thematic analysis “informed by a constructivist paradigm” to understand physicians’ engagement with audit and feedback [52]. Melder and colleagues conducted a longitudinal case study, employed thematic analysis, and examined their themes “through the lens of CFIR” [53]. The point here is to highlight a range of frameworks as ways to extend our learning about D&I by helping researchers to have a rich, nuanced understanding of how interventions are adopted and implemented in diverse settings. By capturing the complexities of real-world implementation, these frameworks help researchers identify critical factors that influence success, inform the design of more effective interventions, and ultimately enhance the impact of implementation efforts.

### The relationship between data collection and data analysis

In D&I research, navigational assistance is helpful in guiding researchers through the choices they face, particularly in the areas of data collection and data analysis. A primary focus of this guidance is on data collection: the foundational methods and techniques used to gather the necessary data. In D&I research, this process involves selecting appropriate instruments, designing data collection protocols, and ensuring the trustworthiness of the data obtained [54]. Data collection can include a range of activities, such as interviews, focus groups, observations, surveys, and the use of existing data sources, among many other methods. The goal is to provide a comprehensive toolkit that researchers can utilize to gather robust and meaningful data tailored to their specific research questions.

Data analysis plays an integral role in the research process. Data analysis is the methodical examination of data to uncover patterns, relationships, and insights that address the research questions. In D&I research, this often involves both quantitative and qualitative methods, such as statistical analysis, thematic analysis, and mixed methods approaches. The nature of the data collected directly influences the choice of analysis techniques. For example, focus group data yields analysis at the group level of analysis; observational data may allow for analysis of actual (rather than reported) behavior. In this way data collection and data analysis are inextricably linked. Therefore, understanding the interplay between data collection and data analysis is essential for researchers to draw valid and actionable conclusions.

The distinction between data collection and data analysis lies in their respective roles and processes within the research framework. Data collection aims to gather raw data relevant to the research questions through designing instruments, selecting samples, and executing data-gathering activities, resulting in raw data such as survey responses, interview transcripts, observational notes, and existing datasets. In contrast, data analysis aims to interpret and make sense of the collected data to answer research questions by applying statistical or thematic techniques to analyze the data, identify patterns, and draw conclusions, resulting in processed information, insights, and findings that address the research objectives.

The navigational assistance provided here will focus on data collection, i.e., ways to get the data, with less emphasis on analyzing the data that results from collection. Yet, analysis is an important means to answering research questions and is not divorced from the way data are collected, as noted above. However, data do not exist to be analyzed without having them first. Therefore, we do offer some assistance to begin thinking about analysis. Common groupings of qualitative data collection methods include elicitation, observation, and document/artifact review methods. Common groupings of qualitative data analysis methods include the broad categories of inductive, deductive, and hybrid approaches that are reflected in more commonly known approaches. Table 1 provides an overview of these methods groupings. Further detail on specific types of methods within these groupings is provided in Tables 2 and 3. Table 4 provides an overview of data analysis considerations.

**Table 1:** Data Methods Groupings

Data Collection Methods Groupings
Elicitation (e.g., via interviews, photovoice) – Focused on having participants share in words, writing or other forms (e.g., photos) their experiences, impressions, descriptions, etc. Observation (e.g., via participant observation, ethnography) – Focused on watching participants’ behavior and actions, and/or observing the artifacts of communication or media Document/Artifact (e.g., via archival analysis) – Focused on data in written or other captured form
Data Analysis Methods Groupings
Inductive – Focused on identifying emergent themes from the data Deductive – Driven by a *priori* research questions that are explored through the research Hybrid – A blend of inductive and deductive Mixed – Integrating different types of data in an intentional fashion, usually driven by a *priori* research questions

**Table 2:** Elicitation data collection methods considerations

**Decision Considerations**	**Structured**		**Semi-Structured (SS)**							**Unstructured**
	**1:1 Interview**	**Time Driven Activity Based Costing**	**1:1 Interview**	**Dyadic (2 respondents)**	**Group-Based**	**Cognitive Task Analysis (CTA) and/or Process Mapping (PM)**	**Critical Incident (CI)**	**Concept Mapping (CM)**	**Written elicitation**	**Standard**
**Brief Description**	Individual interview with a single participant using a predetermined set of questions or topics (e.g., interviewer asking the same set of questions to each participant in the same order)	Individual or small group interview that completes cost accounting that integrates the element of time as a central factor in cost allocation (e.g., cost of resources such as labor, equipment, and facilities per unit of time)	Individual interview with a single participant using a predetermined set of open-ended questions or topics as a guide (e.g., interviewer asking questions to a patient and following up with additional questions and probes based on the patient’s responses)	Paired or dual interview where participants are interviewed together by a single interviewer (e.g., interviewer asking questions to a couple or parent/child)	Group interview modification in which techniques and interventions are used that involve interactions among multiple individuals within a group setting (e.g., focus groups, public deliberation groups, community-based participatory research)	Methodologies used to understand and improve processes, tasks, and workflows to provide comprehensive insights into complex systems (e.g., think-aloud protocols, observation, interviews, diary studies)	Techniques used to gather detailed information about specific events or situations that have significant importance or impact (e.g., gaining deeper insights into human behavior, decision-making processes, and organizational dynamics)	Using tools to visually represent and organize knowledge, ideas, and relationships between concepts (e.g., graphical representations of concepts and their interconnections that help individuals to clarify their understanding, communicate complex ideas, and identify patterns)	Techniques used to gather information or elicit responses from individuals through written means (e.g., open-ended surveys, brain writing, essays, diaries, or journals)	Can be individual or group in which the interview adheres to some standard principles or guidelines, yet allows for flexibility and exploration that often capture rich, detailed insights (e.g., appreciative inquiry, person-centered, phenomenological)
**Conceptual**										
Need to develop understanding	Less so as questions only have mostly set answer options, some explanation can be provided	Good for identifying specific costs associated with tasks and new interventions or implementation strategies	Good for ability to ask privately depth questions and probe for understanding	Good for ability to ask questions of a small group and get small group consensus	Good for ability to get at “group think” and understanding	Good for detailed understanding of how processes work and what people think about them (if CTA)	Good for in-depth understanding of a phenomenon and how it occurs, what the participant was thinking and feeling	Good for uncovering conceptual issues, belief systems, mental models	Good when participant wants to narrate and writing works – gives time to think and process and correct themselves	Good for broad general understanding for exploratory work
Need to verify fidelity/accuracy/presence of behavior	Interview methods are not ideal for this, more for participants’ perception of the behavior process, etc	Concrete tasks can be listed and quantified which can help with documenting actions; recall bias can be a concern	Reliant on participants’ accuracy and honesty which may be unreliable	Same as other interview methods; given others in the group, participants may not want to reveal lack of desired behavior to the others	Reliant on participants’ accuracy and honesty which may be unreliable; participants may not want to reveal lack of desired behavior to the group (social desirability bias)	Given the steps involved, more likely to recall accurately the steps in the process (especially if repeated) and allows ability to explain	Same as other interviews	Given the method of drilling down to concepts, this method is inherently good at revealing inner thoughts; not strong at describing actual behavior	Given the presence of writing, may encourage either more or less honesty in responses; still susceptible to issues of interview methods	Same as other interview methods
Need to consider context in the inquiry	Less so because questions and types of answers are set in advance, unless already included	Less so unless the context is who is playing what role and the resources needed	Allowance for participants to explain circumstances, thus context can emerge and be described with other factors	Same as other interviews, except get the small group perspective	Same as other interviews except get the group perspective	To the extent that workflows are context, this would be included	Participants can explain how the incident occurs within their context	Participants can explain how their beliefs, mental models, etc. interact with context	Same as interviews, but may allow for less probing by the researcher	Yes, quite a bit if featured in the participant’s response and researcher’s probing
**Practical**										
Expertise on the team	Higher level expertise needed to develop the guide and less experience to conduct the interview since it is following a very specific script	Expertise in conducting this type of data collection by guide developer with training for the interviewer is needed	Developer needs expertise to develop good guide with standard interview techniques; interviewer needs training and ability to know when/how to probe. For specific types, need more experience in those methods	In addition to SS, expertise in facilitating multiple respondents in a small group session	To handle skillfully, need experienced facilitator(s) to facilitate a large group of respondents	In addition to SS, knowledge of CTA and/or PM methods for developer and interviewer	In addition to SS, knowledge of CI methods for developer and interviewer	In addition to SS, knowledge of and training in conducting CM interviews; experience with using C-Maps software	Same as SS interviews; knowledge of how to ask good questions via written form	Higher level of expertise to know when to probe, about what and how
Time and availability of team members	For the data collection itself, depends on the time needed; this may be shorter based on length of questions/answers	Usually takes a minimum of an hour or more depending on tasks and number of staff involved	Depending on number and length of interviews, can be more efficient to get to key information than group methods	Depending on the information needed, may take more time to cover the same amount of info than 1:1 to allow all to answer fully	Same amount of time for the participants, but less time than the same participants 1:1 for the interviewer(s) but more setup time; usually need two facilitators; more preparation time	Usually takes a minimum of an hour, up to 2 h. Can do individually or in a small group of participants	Depending on what and how many incidents but usually plan for at least 30 min to an hour	Conducted with one participant. Helps to have two interviewers, one for the mapping and the other to ask questions/probing	Same as other interviews unless participants are slow writers. May be able to be conducted over time in several sessions	Variable in time depending on the key question (topic of the interview), depth of detail needed and verbosity of the participant
Guide development	Need to develop all questions and answer choices like a survey	Involves preparing specific tasks to cover and time for each staff member; usually includes an Excel document that gets completed	Need a guide with key questions and probes	Need a guide with key questions and probes and guidance involving participants	Need a guide that includes key questions as well as facilitator probes to elicit from different participants	Involves key questions as well as process outline for the steps; may require pre-work to develop a sample map for the PM for participants to react to/correct; CTA involves steps on separate papers	Need guide to outline steps of the incident; include the four key questions for probing	Need a guide to identify the beginning question and have appropriate probes; examples to show participant and linking word ideas helpful	Need stem questions for participants to react to in writing	Less time since there is very little guide development; mostly 1–2 key questions with probe areas; could develop list of topics to explore
Resources and other preparation (same for all if providing incentives; same for all if videoconferencing then that is needed)	Answers mostly set from responses, so transcription likely not needed	Usually completing an Excel form with set answers; some transcription needed for narrative portion	Transcription recommended depending on analysis plans	Transcription recommended depending on analysis	Flipchart/use of white board, sticky pads, markers (if in person); online white board, other tools if online; transcription may be needed depending on analysis	Use of process mapping software or other software; sheets of paper for steps or with flow diagrams drafted; may be transcribed	Transcription recommended depending on analysis	Use of the C-Map software program; often recorded but not transcribed	Paper/writing utensils or computer to complete the writing; transcription not needed	Transcription likely required due to open-ended nature of data
Logistics and Access/burden to researchers and participants	Similar burden across types given the same time constraints; consider if follow-up conversation is needed; access issues vary by other factors	Same	Same	Small groups can take longer to coordinate and more time to set up; less time for researchers, same amount of time for participants as 1:1 interviews	More difficult to coordinate than individual interviews unless an already existing group; less time for researchers to do just the group, same amount of time for participants as 1:1 interviews	Same	Same	Same	May be easier for participants to complete asynchronously, thus less time for researchers	Same

*Abbreviations:* Critical Incident (CI), Concept Mapping (CM), Cognitive Task Analysis (CTA), Process Mapping (PM); Semi-Structured (SS)

**Table 3:** Observation and document-based data collection methods considerations

Decision Considerations	Observational Methods					Artifact or Document Methods		
	**In person Observation**	**Virtual Observation**	**Participant Observer**	**Recorded Observation**	**Photo Voice/Digital Story Telling**	**Social media**	**Other Documents**	**Medical or Public Health Records**
**Brief Description**	Direct observation of behaviors, interactions, and environments in real-time at the physical location where the activity occurs, providing rich contextual insights Public places and venues	Observation conducted through online platforms or video conferencing tools, allowing for remote monitoring of behaviors and interactions in digital environments Zoom meetings or classes (with or without recording)	The researcher actively engages in the environment or activities being studied while simultaneously observing, blending insider perspectives with analytical observation Surprise patient, participant in meeting or class	The use of audio or video recordings to capture events or interactions for later analysis, enabling detailed review and the potential for multiple analyses by different observers Encounters video-recorded and then participants reflect on videos (video-based reflexive ethnography)	Participants capture and share their experiences through photographs or digital narratives, offering visual and personal insights into their lives or communities Equipping participants with technology to express their perspectives	Analysis of posts, comments, and interactions on social media platforms to gather data on public opinion, behaviors, and social trends Chat rooms, social media, apps	Review and analysis of various written materials, such as reports, letters, or organizational documents, to understand historical contexts, institutional processes, or communication patterns Curricula, policies, organizational documents (mission, annual reports, meeting minutes, etc.)	Examination of clinical records, health reports, or public health databases to collect data on health trends, treatment outcomes, or epidemiological patterns Electronic health records, court records
**Conceptual**								
Need to develop understanding	Ability to see what is happening, but not ask questions about it (may be liable to misinterpreting reasons)	Ability to see what is happening, but not ask questions about it (may be liable to misinterpreting reasons)	Ability to see what is happening and experience from the “insider view,” may or may not ask questions about it (may be liable to misinterpreting reasons)	Ability to see what is happening, but not ask questions about it (may be liable to misinterpreting reasons)	May enhance understanding “in the moment” by deeply engaging participants in their own context, but may require explanation (combine with interview)	Ability to understand to the extent that rationale is revealed in the context available	Ability to understand to the extent that rationale is revealed in the context available	Highly contextual and usually short text so may be difficult to interpret without additional methods like an interview; need to utilize a skilled person to interpret
Need to verify fidelity/accuracy/presence of behavior	Best at fidelity confirmation	Very good at fidelity confirmation for some circumstances (in person or recorded in person better for some)	Better at fidelity through experiencing it personally but may be fallible to observer bias	Best at fidelity confirmation; allows ability to review multiple times to enhance precision of review and confirmation of observation	Can have participants document their reality to verify	Depending on what is being assessed, more like an observer method to verify behaviors/issues	May have significant gaps in what is assessed, but can determine fidelity in some cases, although this is still documentation of what happened rather than observing it happen	Generally limited in what is provided; a means of assessing what was documented, not what exactly happened
Need for participants to have voice	Allows demonstration but not voice	Allows demonstration but not voice	Allows demonstration but not voice, except participant can share their experience	Allows demonstration but not voice	Excellent as participants get to choose what to capture within the request	Only what documents provide, which might be missing key components	Only what documents provide, which might be missing key components	Only what is documented by the provider/personnel, so participant voice missing
Need to consider context	Allowance to see context in action	Allowance to see context in action, although some lacking in some cases due to remote	Allowance to see context in action, although may miss some aspects while participating	Allowance to see context in action, although some features may be missing from the recording versus in person	Can be excellent if participants can capture this through photos and videos	May not be as likely with online review only	May present limited view of context	May not include context unless documented in notes
**Practical**								
Expertise on the team	Skilled person needs to develop the template for assessment and guide the process, team members can follow	Skilled person needs to develop the template for assessment and guide the process, team members can follow	Need trained person with ability to be a participant (characteristics) and play two roles (participant and observer)	Someone with technical skills to record and set up at the site and then review with participant(s) later	Need to know how to prompt what is requested so that meaningful pictures and videos come back	Training and ability to access social media relevant to the study	Expertise in obtaining and gathering relevant documents	Access to records; expertise in interpreting what is in them
Time and availability of team members	Can be time consuming depending on the amount and length of observations	Can be time consuming depending on the amount and length of observations	More time consuming depending on length and number of participants’ experiences sampled	Less time consuming if review is done efficiently (in a targeted fashion)	Less burden for team members as participants do the picture and video taking	Depends on the availability, access and duration of materials; may need to be available at certain times but may be asynchronous	If enduring, can be more flexible time and amount of time depends on number and length of documents	Gaining approval and access can be time consuming as well as having expertise to extract desired information
Budget available and resources needed (all)	Observation is not usually paid so can be less expensive, although travel to the site can be costly if in person	Observation is not usually paid so can be less expensive; travel not required usually	Observation is not usually paid so can be less expensive, although travel to the site can be costly if in person	Cost to purchase equipment for recording may be necessary	Need to use personal phones or provide camera/video equipment; usually pay incentives for interview portion	Not usually a cost unless accessing materials that need to be paid for, such as subscription-based content	Not usually a cost unless accessing materials that need to be paid for	May be a cost to access the records; may need to pay experts to extract and interpret
Turnaround time	Depends on if the events to be observed need scheduling in advance or are happening regularly	Depends on if the events to be observed need scheduling in advance or are happening regularly	Depends on if the events to be observed need scheduling in advance or are happening regularly, difficulty of joining in/advance notice	Similar to observer methods, may need time to set up and get approval first	Depends on participants’ time schedule and promptness	Less lead time in most cases if already available digitally	Less lead time in most cases if documents are already available	Accessing documents can take approval and time to access through expert
Access and burden to participants	Unless site staff need to assist, less burden to participants; may need to obtain consent if not public place	Unless site staff need to assist, less burden to participants; may need to obtain consent	Unless site staff need to assist, less burden to participants; may need to obtain consent	Unless site staff need to assist, less burden to participants; consenting participants takes time/approval	Participants spend more time doing this; consent process	Usually not an issue in most cases as not involving participants actively; consent may be needed for some cases	Usually not an issue in most cases as not involving participants actively; consent may be required for some cases	May be difficult to access given above barriers listed
Access to technology and needed materials	Not usually necessary	Need software to access remotely and record in some cases	Not usually necessary unless participation requires something	Need access and permission to record and have recording equipment and know how to use, save, store properly	Need to have the equipment for capturing pictures and videos	Need equipment and access to the sites reviewing	Need access to the documents relevant	Need access to the documents which may require technical equipment

**Table 4:** Data analysis methods considerations

Decision Considerations	Qualitative Only					Mixed		
	**Inductive**			**Deductive**	**Hybrid (can be inductive or deductive)**			
	**Grounded Theory**	**Phenomenology**	**Immersion Crystallization**	**Rapid**	**Thematic Analysis**	**Multiple Matrixed Case Study**	**Configurational Methods (CNA and QCA)**	**Joint Display**
**Brief Description**	A method that involves the construction of theories through the systematic gathering and analysis of data	An approach that aims to describe particular phenomena, or the appearance of things, as lived experience by exploring how individuals make sense of experience and transform experience into consciousness, both individually and as shared meaning	This method involves cycles of deep immersion into the data followed by periods of reflective analysis that help the researcher gain insights. This process continues until a deep, complex understanding of the data is achieved, often leading to synthesis of information	A streamlined approach that involves summarizing raw data, identifying key themes, and making swift interpretations often to quickly draw conclusions and insights from data	This method focuses on identifying, analyzing, and reporting patterns within data by organizing; describes the dataset in rich detail and interprets various aspects of the research topic	This approach uses a comparative, multi-case study framework where data is organized across various matrices to facilitate comparison, contrast, and synthesis across different cases, to understand complex phenomena across multiple settings or groups	These methods are case-based approaches that use set theory and Boolean algebra to identify and analyze complex causal relationships by examining how different conditions combine to produce outcomes	This approach visually integrates quantitative and qualitative data in a single display to facilitate the comparison, validation, and interpretation of mixed methods research findings
**Conceptual**								
Are you using a Theory, Model or Framework (TMF)?	Typically avoids pre-existing TMFs to allow for the emergence of new theories directly from the data, though it can later be compared to existing theories	TMFs can aid in the interpretation participants’ lived experiences; these are usually broad and flexible to capture the essence of the phenomenon	TMF can help to structure the iterative cycles of data immersion and reflection, ensuring comprehensive coverage of relevant concepts	Using a TMF is beneficial for quickly focusing the analysis on key constructs and relationships relevant to the research question	TMF can guide identification and categorization of themes that align with established theoretical constructs or models	TMFs can be used to organize and interpret the analysis more effectively, allowing for comparison of cases across consistent theoretical dimensions	TMFs can guide the selection of conditions and outcomes to ensure that the configurational analysis aligns with established theoretical perspectives	TMFs can guide the integration of qualitative and quantitative data in a joint display, ensuring that the synthesis reflects relevant theoretical constructs and relationships
What is the level of quality and being “right” needed?	Demands a high level of quality through meticulous data collection and constant comparative analysis to develop a robust and credible theory	Requires a deep and nuanced understanding of participants' lived experiences, emphasizing the quality of capturing the essence of these experiences accurately	Necessitates high quality in the iterative cycles of immersion and reflection to ensure that crystallized insights are both comprehensive and authentic	The level of quality needed in rapid analysis is often balanced with the need for speed, prioritizing actionable insights over comprehensive detail	Requires thoroughness and rigor in coding and theme development to ensure accurate and meaningful interpretation of data	Achieving high quality involves detailed and systematic comparison across cases to ensure accurate and reliable conclusions	High quality and accuracy are crucial to ensure the correct identification of complex causal relationships and configurations	High quality is essential to accurately integrate and present qualitative and quantitative data, ensuring a coherent and comprehensive interpretation
Is there a lot known on this topic already? Looking for confirmation of previous results?	Less suited for topics with extensive prior knowledge as it aims to generate new theories rather than confirm existing ones	Best used for exploring new dimensions of lived experiences, making it less focused on confirming existing knowledge and more on deepening understanding	Can be applied to topics with prior knowledge, allowing for the confirmation and elaboration of existing insights through iterative analysis	Useful when there is already substantial knowledge on a topic and quick confirmation or action-oriented insights are needed	Can effectively confirm and build on existing knowledge by identifying recurring themes and patterns consistent with previous research	Suitable for confirming and comparing previous results across different contexts or cases, leveraging existing knowledge to inform analysis	Well-suited for topics with extensive existing knowledge, as they can confirm and elucidate complex causal patterns and interactions	Effective for confirming previous results by visually integrating and comparing qualitative and quantitative data, highlighting consistencies and discrepancies with existing research
Are you trying to assess across multiple sources or see a case-by-case pattern?	Focuses on developing a theory through detailed case-by-case analysis, often resulting in patterns that emerge across multiple sources	Focuses on the deep, case-by-case exploration of individual experiences to uncover the essence of a phenomenon	Allows for both deep case-by-case analysis and the identification of patterns across multiple sources through iterative immersion and reflection	Suited for assessing across multiple sources quickly to identify common patterns and key insights	Flexible and can be used to assess both across multiple sources to identify overarching themes and within individual cases for specific patterns	Specifically designed to assess patterns across multiple sources by comparing and contrasting different cases systematically	Ideal for assessing across multiple sources to identify complex causal patterns and interactions within different configurations	Allows for the simultaneous assessment of data across multiple sources and case-by-case patterns by visually integrating qualitative and quantitative findings
**Practical**								
Do you need the results fast? Or iteratively?	Typically iterative, requiring continuous data collection and analysis to develop a comprehensive theory	Involves an iterative process to deeply explore and understand lived experiences, often requiring more time	Inherently iterative, involving cycles of deep engagement with the data and reflective analysis to crystallize insights	Designed to produce results quickly, making it suitable for situations requiring immediate insights	Can be conducted both quickly for initial insights or iteratively for a more in-depth understanding, depending on the research needs	Can be both fast, for initial cross-case comparisons, or iterative, for thorough analysis and understanding of patterns across cases	Typically require an iterative approach to identify and refine complex causal relationships across cases	Can be used either quickly for initial integration of qualitative and quantitative data or iteratively for deeper and more comprehensive analysis
What is the expertise on the team?	Requires a high level of expertise in qualitative research and familiarity with iterative data collection and coding processes to develop new theories	Necessitates expertise in philosophical approaches and the ability to deeply interpret lived experiences, often requiring specialized training	Benefits from a team experienced in iterative and reflective analysis, capable of engaging deeply with the data over multiple cycles	Suitable for teams with practical experience and the ability to quickly synthesize key insights, often without requiring deep theoretical knowledge	Suitable for teams with a basic understanding of qualitative methods, though more experience can enhance the depth and rigor of the analysis	Requires expertise in case study methodology and the ability to systematically compare and contrast data across cases	Require expertise in set theory, Boolean algebra, and familiarity with configurational comparative methods to accurately analyze complex causal relationships	Benefits from a team with expertise in both qualitative and quantitative methods, as well as skills in data visualization to effectively integrate and present findings
What type of data do you have collected?	May require more in-depth and descriptive data such as through elicitation methods	May require both elicitation and observational methods to explore the depth of the experience	Multiple data collection methods may be used	Multiple data collection methods may be used	Multiple data collection methods may be used	As it is mixed, requires both quantitative and qualitative data	As it is mixed, requires both quantitative and qualitative data	As it is mixed, requires both quantitative and qualitative data
Do you have quantitative data? Or will you convert qualitative to quantitative data?	Typically does not use quantitative data, focusing instead on developing theories directly from qualitative data	Relies on in-depth qualitative data to explore lived experiences and is not generally concerned with quantitative data or conversion	Primarily deals with qualitative data through iterative analysis and does not typically convert findings into quantitative data	Can incorporate quantitative data or convert qualitative findings into quantifiable metrics for quick, actionable insights	Can complement quantitative data by identifying themes that can be quantified for mixed methods analysis, though it primarily focuses on qualitative data	Can integrate both qualitative and quantitative data, using matrices to compare and contrast cases in both forms	Can handle both quantitative data and converting qualitative data into set memberships to analyze complex causal relationships	Designed to integrate and visually represent both qualitative and quantitative data, making it suitable for studies that encompass both types of data or convert qualitative insights into quantitative metrics

*Abbreviations:* Coincidence Analysis (CNA), Qualitative Comparative Analysis (QCA), Theory, Model or Framework (TMF)

## Considerations in decision-making

First, we begin with those “things to think about” before we start into different navigational pathways. Tables 2 and 3 provide the methods groupings with specific data collection methods types and a description and some examples. This is not meant to be an all-inclusive list; however, it should expand thinking beyond simply conducting a focus group! These tables are meant to be a starting point for both practical and conceptual considerations: practical because of limitations in budget, personnel, or other resources to do the work (something is better than nothing) and conceptual relating to what is it that is being studied and how can we best conform to the research question(s) at hand. Sometimes the resources available dictate what can be “on the menu” for methods choices. Sometimes the end result goal precludes some methods choices. It is also worth pointing out that all methods have their strengths and limitations. We encourage being expansive in your consideration of options, rather than constrained or discouraged by limitations, which are inherent to any one method.

**Fig. 2:**
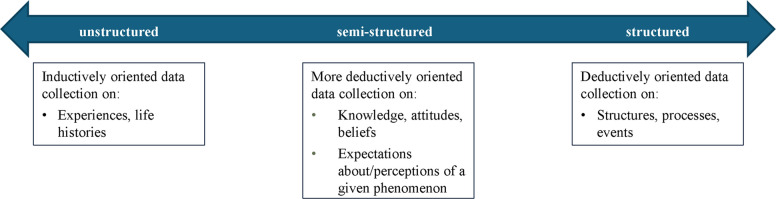
Qualitative Data Collection Spectrum. Adapted from Hamilton AB, Fix GM, Finley EP. Pragmatic Healthcare Ethnography: Methods to Study and Improve Healthcare. Taylor & Francis; 2024 Dec 9

Table 2 outlines considerations for elicitation methods like interviews and focus groups. The table illustrates how these methods differ so researchers can consider the relative merits of each for their own study purposes. There are a few important key points that apply to all. One is that elicitation methods as compared to other methods (Table 3) provide the benefit of allowing voice. This means the participant can narrate their own perspective by answering in their own way within the parameters of the guide. However, considering the continuum from structured to unstructured data collection (Fig. 2), more variability in the latitude of the participants leans toward much more open dialogue in the unstructured elicitation approaches. This can be challenging given their lack of structure but also insightful and rich given their emphasis on the participants’ ways of expressing themselves (i.e., often in unstructured qualitative data collection, a guide may not even be used).

Important to allowing voice is the attention to power dynamics with any type of elicitation method, including consideration of the status and characteristics of the researcher(s) relative to the participants, as well as the composition of the sample (e.g., not convening people at different levels of a hierarchy in a focus group). From a practical perspective, there are also differentiating factors based more on the design and sampling approach rather than the type of elicitation method selected. For example, the difference in turnaround time (time from identifying participants to getting the method completed), logistics, or burden of access for the researcher/interviewer or participants can vary considerably. Focus groups can take a lot more time to plan and organize the schedules of multiple participants (such as busy clinicians) than 1:1 interviews; however, collecting all the information in one 1.5-h period with 10 participants all together can be efficient compared to doing 10 individual 45-min interviews. These approaches yield fundamentally different data (group- versus individual-level data) and are not interchangeable during data collection or analysis. Always, there is a weighing of the bottom-line goal of the project with what is appropriate to address the research question(s). Another factor across all elicitation types is the practical issue of resources and other preparation needed. For example, incentives for participation are generally recommended, if allowable, and should be considered in budgeting. All methods can be completed via video conferencing (e.g., Zoom/Teams or other software, with considerations of access and scheduling) or in person (with considerations of room scheduling, location, accessibility/comfort). Considerations of these details are dependent on the study question as well as feasibility. A limitation of elicitation methods is that all are susceptible to bias since they rely on participants to be knowledgeable, accurate, and honest, as well as be able to explain, using words, their perspectives. The researcher may wish to focus more on observation and other methods for assessing fidelity and verifying behavior [55].

Table 3 outlines considerations for other methods like observation and documents. The table illustrates how these methods differ for researchers to consider the relative merits of each for their own study purposes. Again, there are a few important key points to be made about all, although fewer than elicitation methods because they are inherently more variable. The observation method of photovoice and/or digital storytelling is notably different in that it is actually a combination of a type of observation with a type of elicitation; recent work has highlighted the value of this and other more community-engaged approaches for implementation science [56, 57]. The main benefit of these methods is the ability to verify actual behavior and experience a phenomenon in real time or the residual artifact of it. A limitation of document analysis is the lack of explanation of what is happening and knowing that all the “there is there.”

Table 4 outlines possible data analysis decisions. There are certainly many more options to be explored than presented here, but this is a starting place to expand thinking about analysis methods that can be used in D&I research. As with Tables 2 and 3, considerations are presented including both practical and conceptual. A key decision point is whether the research question is inherently inductive or deductive and if there are quantitative data also available for analysis. D&I research often has more of a deductive nature to it as we utilize TMFs for formulating an answer about what factors are facilitating or impeding an intervention from working in a naturalistic setting. However, we encourage D&I researchers to consider more inductive approaches for the important D&I work of “designing for dissemination” [58] including starting from the very beginning about what might be important factors without preconceived ideas, and working with more inclusive and engaged approaches such as co-creation, [59] user-centered design, [60] “citizen-engaged” implementation science, [61] and the broader field of Patient and Public Involvement and Engagement, [62] as well as Indigenous research methodology [63].

## Navigating methods choices based on the research goal and question(s)

We begin this navigational discussion based on the goal of the research. What are you mainly trying to accomplish? These are divided into different types of goals with respect to choosing a pathway. These include the primary goal of developing something new, adapting an intervention or approach, and learning about/conducting an evaluation of an implemented intervention or implementation or dissemination strategy. As noted above, it is important to start with having good research questions and being clear about what you are trying to accomplish. Then consider both the overall study design as well as how to design the data collection and analysis approaches that will best answer that question, and then consider the specific individual methods that are best employed; in this case we focus on qualitative methods, with some consideration of mixed methods.

**Fig. 3:**
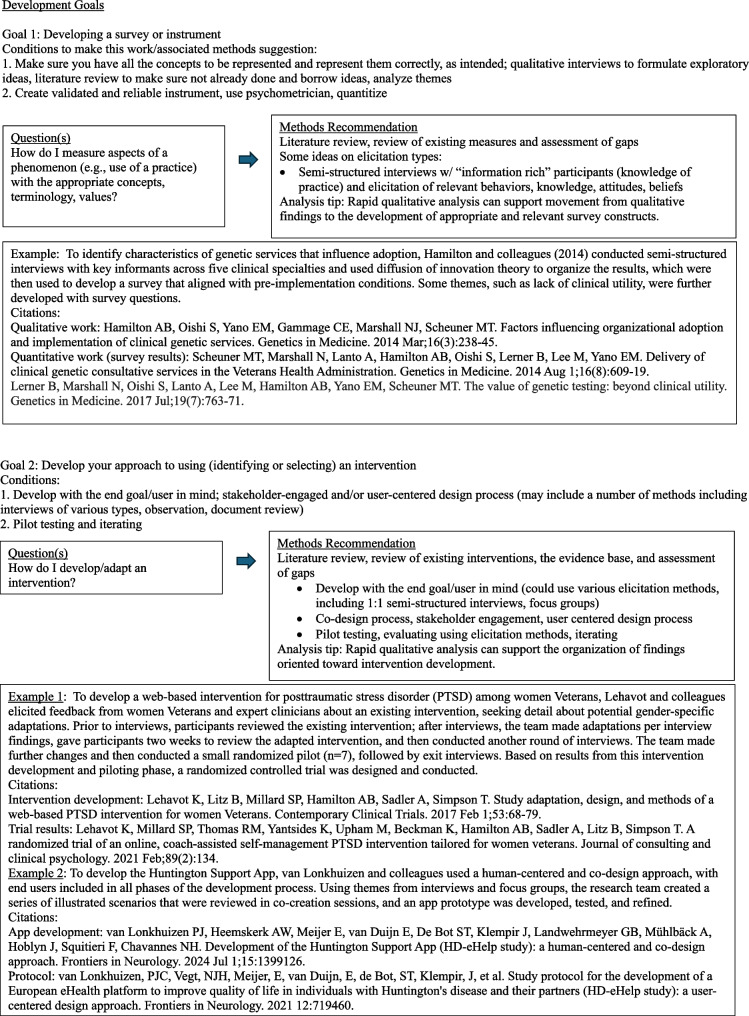

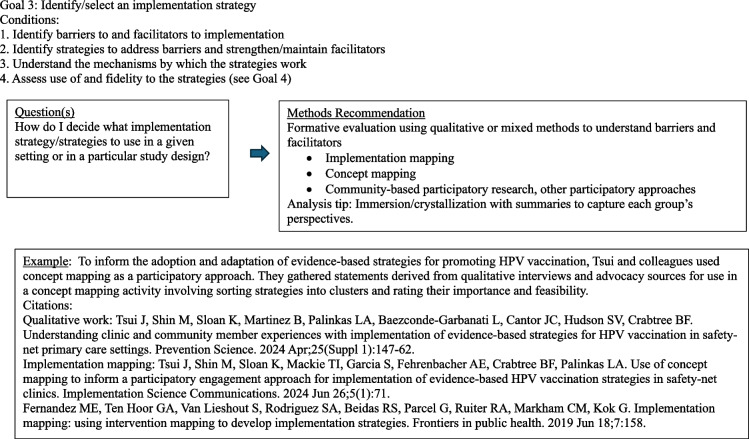

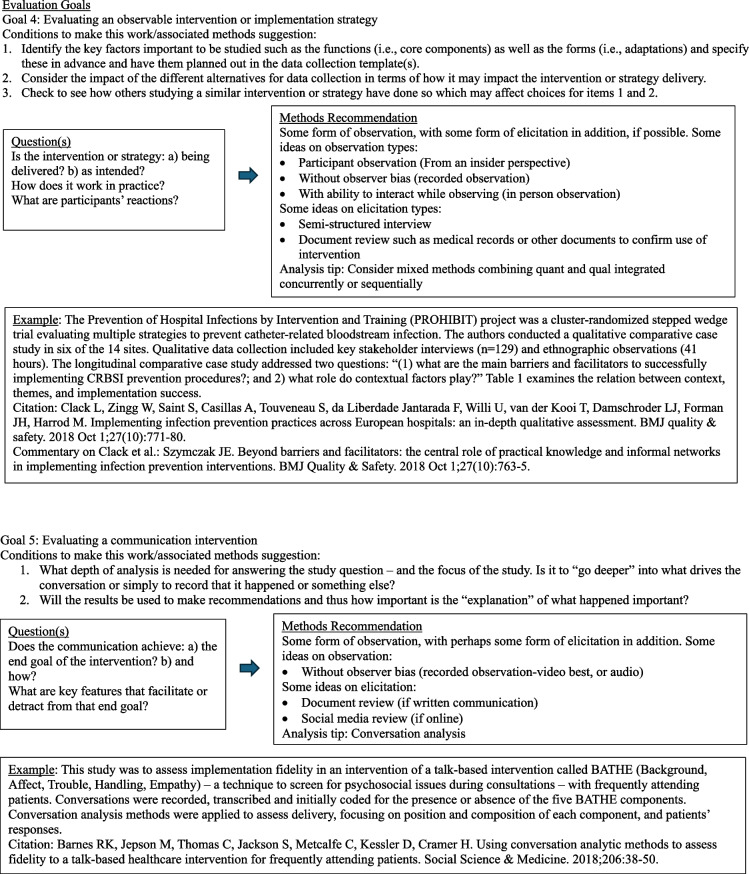

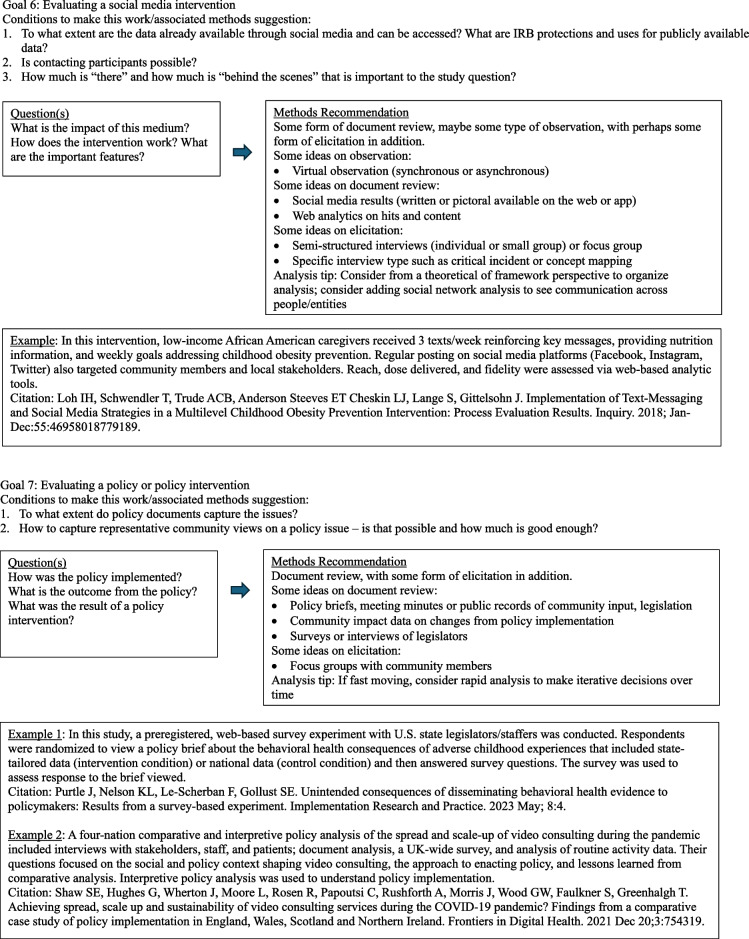

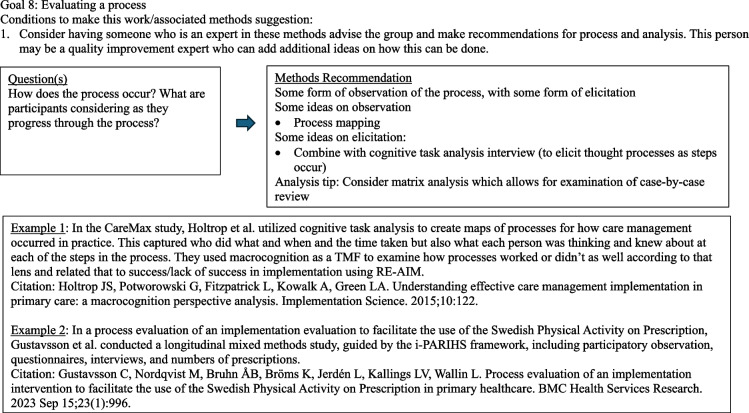
Goal-directed methods suggestions [64–75]

The diagrams under Fig. 3 outline common question groupings and methods considerations that evolve from a question and design. Note that this section is written with guidance in mind. Although this may seem a bit cookbook or plug-and-play, it is not intended as such. The focus here is to encourage the D&I researcher to begin with the end goal in mind and then work backwards with an array of methods choices available. The idea is to get creative and expansive with methods options and not simply rely on interviews. Thus, ideas and examples are provided for consideration among many options. It is important to note that reading this paper does not substitute for deep understanding of qualitative methods. We advocate for the inclusion of methodologists with this expertise in any research.

## Example of methods application in a specific study

PATHWEIGH was an NIH-funded D&I study focused on improving the care for patients with the common condition (and now considered disease) of obesity. Obesity is associated with over 200 known conditions from cancer, heart disease, diabetes, to sleep apnea and musculoskeletal issues such as joint replacement [76]. To meet the need for effective interventions to address obesity, we developed and deployed PATHWEIGH across all adult-serving primary care practices in one large health system (1R18DK127003). PATHWEIGH is not a weight loss program, but a care process to treat obesity and manage weight long-term in primary care. Specifically, it centers around an electronic health record (EHR)-driven care process that includes a specialized visit type, pre-visit questionnaire, smart texts and smart sets to make obesity care more effective and efficient. To support the intervention, implementation strategies were deployed including training, consultation support, and practice champions. PATHWEIGH studied outcomes of weight loss and maintenance (effectiveness) and adoption and implementation (implementation) in a cluster-randomized, covariate constrained stepped wedge cluster design study, with a hybrid type 1 effectiveness-implementation approach [77]. The study utilized the RE-AIM and PRISM TMFs for exploration of the research questions. This study serves to illustrate the navigational assistance we provide throughout the paper.

**Fig. 4:**
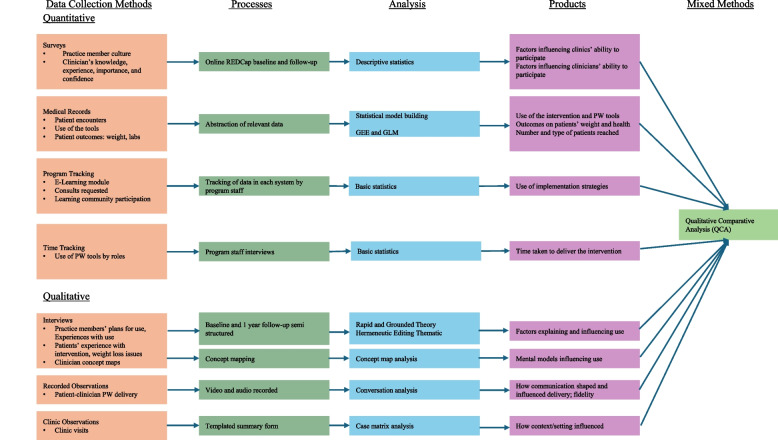
PATHWEIGH example

The quantitative methods (for reference) included surveys, medical record data capture, and claims data. The qualitative methods included interviews (including concept mapping and cognitive task analysis) at the patient and practice team member levels, observations with recordings of patient visits, and review of materials and medical record documents. Qualitative analysis methods included rapid, grounded theory (hermeneutic editing modification), matrix analysis, and conversation analysis. We used the artificial intelligence (AI) option within ATLAS.ti to more efficiently study our data [78]. Finally, we used qualitative comparative analysis (QCA) to put all of the data together to understand the necessary and sufficient conditions that are needed for effective use of the PATHWEIGH intervention in practice. The selection of methods was intentional to discover deeper answers to specific implementation questions about the use and non-use of PATHWEIGH in practice. Concept mapping, described in Table 2, is a type of inquiry and analysis method used to elicit and capture tacit knowledge, including participants’ mental models about complex topics, the results of which are published. The research question for this method was, “What are clinicians’ mental models about care for patients with obesity?” Conversation analysis focuses on how participants accomplish social actions through recurrent interactional practices; [79–81] the results of this analysis were recently published [82]. The research question for this method was, “How did the use of the pre-visit questionnaire influence provider-patient communication about weight in the visit?” Qualitative comparative analysis is described in Table 4 and referenced in several papers by the first author (JSH) [83–86]. The research question for this method was, “What are the key contextual features of practices that had better reach of weight management interventions to patients?” Fig. 4 provides an overview of all the methods used and how they were considered.

This example illustrates how multiple data collection and analysis methods can be combined within one study to examine different phenomena. In PATHWEIGH we learned not only about the weight loss outcome from the intervention but also about how the providers and teams used the intervention components, which ones worked and didn’t, and how features like the clinician-patient communication influenced and interacted with the intervention (recorded observations with conversation analysis), how clinicians’ mental models intersected with the adoption of the intervention (concept mapping interviews with case- based grouping analysis), how the time taken to do the intervention and how it was coded and billed influenced the use (time-driven, activity-based interviews and observations with financial sustainability analysis), and how practical barriers such as full clinical schedules and patients with multiple competing concerns influenced willingness to consider obesity care at all (observations of the practices in real time combined with semi-structured interviews, with rapid and matrix analysis).

## Discussion

This paper was inspired by questions that the authors have fielded over two decades of conducting D&I research with a focus on qualitative methods. Our goal was to characterize a range of options, including but also extending beyond typically selected methods such as individual interviews and focus groups. We also endeavored to illustrate methods decision-making options via eight common goals in D&I research. We reinforce the assertion that data collection choices will affect analytic options, [87] necessitating early and frequent consideration of analytic goals consistent with strong research questions.

This paper adds to the currently available options for learning about qualitative methods for D&I science by expanding on earlier work by the QualRIS group [8] and the work of Hamilton and Finley [5]. More recent work has featured particular methods such as periodic reflections [88] and innovations with rapid qualitative analysis [46, 89, 90]. Scoping reviews have found that ethnographic methods are increasingly being employed in D&I science [91] but that historically, most studies have used a single elicitation design relying on individual interviews or focus groups [7]. This paper complements existing literature by providing navigational assistance, i.e., in what circumstances one might want to consider one particular method over another, or consider blending methods. The goal is to expand the repertoire of methods choices and also to match the method(s) to the research question in a more intentional way.

The paper has limitations in that there are many methods of data collection and analysis beyond those presented here, although we endeavored to cover the main categories of choices. It is likely that the methods presented are those more familiar to the authors, which likely reflects their disciplinary backgrounds. Other backgrounds and training may highlight different methods and examples of how they might be used. However, the authors attempted to include an array of study examples that were not their own and that draw upon a wide variety of D&I study types and content areas. Also, an extensive discussion of how to use the methods is not part of this paper, mostly due to space considerations, but also because other resources exist that accomplish that goal.

Future work in D&I research would benefit from a scoping and/or systematic review of qualitative and mixed methods used across different D&I study designs employing different TMFs in the global implementation context. The recent scoping reviews of contributions of anthropological practice in implementation sciences [92] and of qualitative methods in implementation science [7] serve as excellent models for future explorations of methodological pluralism in D&I research. Additionally, as D&I researchers utilize different methods, we recommend that they include commentary on the selection of the method(s) for the purpose intended, inspired by some journal requirements for articulating the methodological contributions to mixed methods (e.g., *Journal of Mixed Methods Research)*. This could build a community of knowledge about the use (when, how, how well it worked) of various methods such that it might expand the methods researchers are using for their D&I research. This, in turn, could help to accomplish the ultimate goal of better understanding the phenomenon under study and lead to increased and improved use of evidence-based interventions, contributing to optimized health outcomes.

## Conclusion

This paper adds to the field of D&I science by addressing a gap in the existing literature about how to conduct D&I research from a qualitative methodological perspective and by expanding the repertoire of methods choices and how they may be utilized and combined, as well as illuminating areas of thought that would benefit from further exploration. It is the hope of the authors that the information contained here may serve as a guide for D&I researchers and for D&I methodologists in providing consultative assistance.

The joy of conducting research is to discover new understandings and apply them in ways that will benefit others. We have a duty to utilize methods that holistically explain what is happening, why, and how we might plan future endeavors based on new knowledge. Use of more, different, and the most appropriate methods can contribute to this goal. It is also likely that this will prove more stimulating for the researchers themselves.

## Data Availability

Not applicable.

## Abbreviations

AI: Artificial Intelligence
CFIR: Consolidated Framework for Implementation Research
CI: Critical Incident
CM: Concept Mapping
CNA: Coincidence Analysis
CTA: Cognitive Task Analysis
D&I: Dissemination and Implementation
EPIS: Exploration, Preparation, Implementation, Sustainment
PM: Process Mapping
PRISM: Practical Robust Implementation and Sustainability Model
QCA: Qualitative Comparative Analysis
SS: Semi-Structured
TMF: Theory, Model, or Framework
